# The Metropolitan Hospital Sunday Fund Crisis

**Published:** 1912-12-28

**Authors:** 


### The Hospital

The Workers' Newspaper of Administrative Medicine and Institutional Life, Administration, National Insurance and Health.

No. 1382, Vol. LIII. SATURDAY, DECEMBER 28, 1912. PRICE ONE PENNY.

### THE METROPOLITAN HOSPITAL SUNDAY FUND CRISIS.

Those of our readers who have followed year by year the reports of the annual meetings of this Fund which have been published in The Hospital,* however much they may regret it, will be prepared for the position revealed at the annual meeting at the Mansion House, on Friday, 20th instant, reported on pp. 356-8. Last year, in our issue of December 30, 1911, p. 322, we insisted that the Metropolitan Hospital Sunday Fund could not continue to prosper or even to endure much longer unless it had behind it the confidence and good will of the clergy and ministers, of the representatives of their congregations, of the contributors, and of the public at large. We insisted that this meeting of constituents should be so conducted as to give the fullest and fairest opportunity to every accredited representative to take part in the discussions. To hamper or render impotent independent speakers by raising technicalities, and to confine them as much as possible to the routine resolutions submitted on the authority of the Council, must prove a fatal policy.

This year a forgetfulness on the part of the Secretary of a notice handed in by the Rev. Lionel Lewis last December, with copies of resolutions, the receipt of which the Secretary acknowledged and promised should be attended to, created a difficulty. The Secretary issued the notice summoning the meeting for the 20th instant without notifying the resolutions of which Mr. Lewis had given notice. It was held by counsel, when the error was discovered, that, a special notice of a proposed motion for making so drastic a change in the Constitution, as suggested by Mr. Lewis, ought to be given to the constituents before the meeting is held at which it is proposed to submit the motion. Counsel therefore expressed the opinion that Mr. Lewis's motion should not be put by the Chairman, and, indeed, that the Chairman should not allow Mr. Lewis to move it. The Chairman of the meeting, Alderman Sir John Bell, admitted that Mr. Lewis had cause for complaint, especially as he understood that the letter containing the notice had not only been mislaid, but apparently lost sight of. He, however, expressed himself as helpless in the matter, seeing that he was bound by the Constitution of the Fund. He further refused to give a promise that if a second notice of the resolutions was sent in by Mr. Lewis, a special meeting should be called to consider them within two months. This ruling of the Chairman created great dissatisfaction amongst those present, several of whom were the clergy who had attended with their churchwardens in order to support the Council. As one speaker put it, although he came prepared to support the Secretary of the Fund through thick and thin, he must insist strongly that unless some improvement were introduced into the management of the Fund it was liable to be ruined. Many appeals were made to the Chairman to meet the special circumstances which had arisen by affording an opportunity to the constituents of the Fund to assemble in special meeting, that the business ruled out of order might be fully considered and dealt with on its merits. But, as the Chairman declared himself unable to do other than follow exactly the opinion of counsel, the meeting broke up.

Everybody who reads the full report of the proceedings published in another column will realise the seriousness of the crisis which has arisen. This is the sixth consecutive year in which dissatisfaction has been created amongst an increasing number of the constituents by the absence of proper by-laws regulating the procedure at these meetings. The only regulations, if such they can be called, at present published are the Laws of the Constitution. Number 1 of these Laws provides that "the congregations which have forwarded contributions to the Fund during two successive years shall be entitled to a voice in the management of the Fund during the year next following the last of such contributions. A minister and two laymen representing every such contributing congregation shall be summoned as forming the constituents of the Fund, to meet the Council in the month of December in each year, to receive the annual report of the Council for the year and to elect the Council for the succeeding year." That is,

* December 21, 1907, p. 327; December 26, 1908, p. 337; December 25, 1909, p. 309; December 24, 1910, p. 391; and December 30, 1911, p. 341.

we believe, all there is, and if so, this Law requires amendment or supplement. The Fund at present has no clear rules of procedure for the conduct of its business at the annual meeting. The constituents have become in consequence increasingly restive because an impression has been created in their minds that the Constitution as at present drawn and administered does not give the contributing congregations and their representatives the necessary facilities, impartially and fully, to state their views in regard to the distribution of the funds collected. It is contrary to the public interest and must prove fatal to the continuance of the Fund that the representatives of contributing congregations should longer remain dissatisfied, as they justly may be, with the present state of affairs.

The Council cannot fail to appreciate that, after all, he who pays the piper commands the tune. Should ever the clergy and ministers of all denominations, and the contributing congregations throughout the Metropolis so decide, they have it in their power to withhold their contributions altogether, and even to suspend Hospital Sunday itself. It is lamentable that this great Fund should be imperilled, as it undoubtedly is imperilled at the moment, by the absence of business procedure guaranteeing its constituents from the hampering of their freedom of action through mere technicalities such as those which led to the regrettable proceedings at the Mansion House on Friday week.

